# The relationship between mentoring on healthy behaviors and well-being among Israeli youth in boarding schools: a mixed-methods study

**DOI:** 10.1186/s12887-015-0327-6

**Published:** 2015-02-15

**Authors:** Maayan Agmon, Cheryl Zlotnick, Anat Finkelstein

**Affiliations:** The Cheryl Spencer Department of Nursing, Faculty of Social Welfare and Health Sciences, University of Haifa, 199 Aba Khoushy Ave Mount Carmel, Haifa, 3498838 Israel; Administration for Rural Education and Youth- Aliya, Ministry of Education, 2 Hashlosha St, Tel- Aviv, Israel

**Keywords:** Boarding school, Mentoring, Physical activity, Heath habits

## Abstract

**Background:**

Although 10% of Israeli youth live in boarding schools, few studies, except for those focusing on mental health, have examined the well-being of this population subgroup. Thus, the aims of this study were to explore: (1) the prevalence rates of five aspects of well-being (i.e., healthy habits, avoidance of risky behaviors, peer relationships, adult relationships, and school environment) in youth residing at Israeli boarding schools; (2) the relationships between youth well-being and youth perception of their mentor; and (3) the different subgroups of youth with higher rates of risky and healthy behaviors.

**Methods:**

This study used a mixed-methods approach including a quantitative survey of youth (n = 158) to examine the association between youth behaviors and perception of their mentor; and a qualitative study consisting of interviews (n = 15) with boarding school staff to better understand the context of these findings.

**Results:**

Greater proportions of boarding school youth, who had positive perceptions of their mentor (the significant adult or parent surrogate), believed both that their teachers thought they were good students (p < 0.01), and that they themselves were good students (p < 0.01). This finding is supported by the qualitative interviews with mentors. Youth living in a boarding school had very similar healthy habits compared to other youth living in Israel; however, youth in the general population, compared to those in the boarding schools, were eating more sweets (OR = 1.39, 95% CI = 1.02-1.90) and engaging in higher levels of television use (OR = 2.64, 95% CI = 1.97-3.54).

**Conclusions:**

Mentors, the significant adult for youth living in residential education environments, have a major influence on school performance, the major focus of their work; mentors had no impact on healthy behaviors. Overall, there were many similarities in healthy behaviors between youth at boarding schools and youth in the general population; however, the differences in healthy habits seemed related to policies governing the boarding schools as well as its structural elements.

## Background

Parents determine the social environment, health habits and emotional atmosphere for their families. These elements influence well-being and health outcomes of their children later in life [1,2]; however, some children grow up outside the family home without the guidance and supervision of their parents. Many studies have contributed substantial research on the impact of significant adults on youth living outside the family home [3-9]. These studies generally have concentrated on very specific negative outcomes such as risky behaviors and mental health problems, rather than on a broader spectrum of outcomes related to youth well-being and physical health.

Youth well-being is comprised of five parameters: healthy habits, avoidance of risky behaviors, peer relationships, adult relationships, and school environment [10-12]. Healthy habits include diets rich in vegetables and fruit, lacking in refined sugars, and with less saturated fat [13,14]. An equally important contributor to healthy habits for youth is at least one hour of physical activity daily [14]. Yet, in western society, diets are influenced by the pervasive availability of fast food [15]; and physical activity increasingly is being replaced by screen use (i.e., cell phones, tablets, computers, video games, television) [16]. Additionally, youth are engaging in unhealthy diets and low rates of regular physical activity, and as a result are exhibiting higher rates of obesity and early manifestations of chronic diseases such as hypertension and diabetes type II [14].

The social environment, consisting of peers, parents and school, is equally important [11]. For example, youth engaging in risky behaviors, such as drug and alcohol use, are more likely than others to have a weak connection with society, which in turn, increases the risk of: being a school dropout, unstable employment, lower socioeconomic status, and experiencing early mortality [17,18]. Peer relationships are an important component of the social environment, as youth are in the developmental stage of building their self-identity and exploring their position in society [12]. Parents and other significant adults hold an equally essential position in the social environment, as they are the guides and supporters that assist youth to develop independence, confidence and autonomy [12,19]. Lastly, the social environment includes the school. It is the most stable, non-family social context for youth, and the milieu where youth identify friends, gain exposure to both healthy habits and risky behaviors, and gain confidence based on school performance [19,20]. However, these constructs are based on the general population of youth, and rely on the assumption that most youth grow up living with parents. Yet, some youth subgroups do not. Instead, they live in group homes or boarding schools or other institutional sites. This study focuses on one of these subgroups comprising mostly vulnerable youth.

### Health habits and risky behaviors among youth growing Up in Non-Family Residences

Most literature on vulnerable youth living in institutions or residences outside the family home focuses on samples of youth living in foster care or group homes. Youth in these environments are at higher risk for obesity and other health problems [21], risky behaviors [22], poorer peer relationships, fractured adult relationships [23], social distress [24] and dropping out from school [25]. Adult outcomes of youth living in foster care, compared to other youth, have been linked to rates of poorer adult outcomes in educational attainment [23,26], risky substance abuse problems [27], homelessness [27,28], problematic family situations [29] and mental and physical illness [30,31].

The living environment in adolescence influences adulthood health. Youth living in more structured and organized family environments are more likely to possess healthier habits including better diets [1,2,32], regular physical activity [33] and lower rates of substance use [34]. Similarly, family structure in adolescence is associated with lower rates of emotional distress later in life [35]. However, most studies examining the association of living environment and youth outcomes employ samples of youth from the general population. Far fewer have examined the influence of the structured living environment on healthy habits in youth residing in non-family situations such as foster care, group homes or boarding schools.

### Significant adult or mentor

For many youth living at home, the significant adult is a parent [36]; however, for youth living in boarding schools or group homes, the significant adult, may be a non-family member who has a consistent relationship that has developed through a structured program or through other connections [6,8,37]. A systematic review has offered a variety of definitions for mentoring – including providing instrumental and emotion support, having a sustained relationship, and assisting with progressively more complicated life situations [38]. Relationships with an adult mentor lasting longer than one-year have the greatest benefit [4]. Youth who define themselves as having a mentor were less likely to smoke marijuana or become involved in delinquency [9]. They also tended to suffer less than their peers from depression [7]. Moreover, youth with adult mentors showed greater levels of life satisfaction and were more likely to complete high school and attend college compared to youth without mentors [3]. Few studies have examined the influence of mentoring on health behaviors; however, one study on foster care youth found long term mentoring was associated with lower stress [5]. More studies are needed to assess these relationships and develop information on where and how youth living in foster care, group homes or other institutions find mentors or significant adults that match their needs and personalities, and assist and support them as they grow into adulthood.

### Israeli boarding schools

Residential education programs (i.e., boarding schools) is the source of education for more Israeli youth than youth in any other country [39]; at least 10% of youth between age 13–18 live and are educated at boarding schools [40]. The local name for boarding schools is “Pnemia”, meaning “living in”; and these boarding schools possess a model unique to Israel [41]. The boarding school population contains students, who would ordinarily be attending regular schools, but are placed in the boarding school either: to assist with the cultural transition of being a new immigrant to Israel, or due to family challenges such as poverty [42].

Boarding schools usually have onsite educational facilities, living facilities, cafeterias and kitchens, and offices for some professional staff. It is a compound comprising several buildings and recreational areas, including the school and dormitories. The overall operation of the boarding school compound is overseen by the manager or director. Reporting to the director is the “mother of the house”, who is responsible for the day-to-day operation and in charge of the mentors. Mentors are the significant adult or parent surrogate for the youth, and provide 24 hour-7 day per week supervision. Mentors are assigned a group of youth based on the dormitory or living quarters. Within the living quarters, youth are divided by age groups and gender. Usually six youth of the same gender share a bedroom; and groups of approximately 12 to 15 youth share a common room or living room where they gather and meet with their mentors every evening. Teachers at the boarding school are like teachers at any other school. Their responsibilities are solely to provide education to the students.

Policy recommendations for diet, physical activity and other areas of daily living are provided by the Ministry of Education under whose jurisdiction approximately 85% of these boarding schools fall. Among these policies is that youth should visit their families every other weekend at home. Most youth are referred to the boarding school by a mutual decision between social workers and parents. Youth admitted to the boarding schools are characterized by levels. Level one denotes the highest risk level and is assigned to youth who either: are orphaned by one or both parents, exposed to domestic violence, were living with non-functional families, suffered emotional and/or physical rejection by the family, have a police record, are from families of extremely low socioeconomic status, have no contact with community, or are from dangerous environments. Level two is signified as being from: a one-parent family with little parental supervision or weak parental authority, a family with low socioeconomic status, and being at risk of dropping out of school. Level three includes parents who work most of the day, have a low socioeconomic status, provide very little parental supervision, and whose youth need education support.

Within the boarding school staff, there are two types of professional staff, those who live in the boarding school and those who do not. The professional staff living in the boarding school include: 1) the manager or director who is responsible for the operation of the boarding school compound and the safety of its youth, but generally has no daily direct contact with the youth; 2) the mother of the house who is responsible for the day-to-day operation of the boarding school compound, in charge of the mentors, responsible for food, clothes, daily problem solving, and youth who cannot go back to their homes; 3) mentors, who function as parent-surrogates, including providing emotional support and guidance, at least weekly one-to-one meetings and daily group conferences, and close supervision of daily activities who remain in constant contact with the youths’ families and school teachers (usually there are two mentors, one male and one female to 15–20 youth of each age group, who alternate to be onsite 24 hours per day); and 4) mentors’ supervisors are more experienced mentors and available for consultation on situations such as youth behavioral concerns, emotional support and technical problems. The professional staff who do not live on site include: 1) the nurse who is charge of all medical care including dispensing routine medication, immediate triage of injuries, determining the need for physician referral, daily consultation for health concerns, monitoring health status (e.g., height, weight, blood pressure) and providing vaccinations; 2) the social worker who is responsible for providing guidance on social problems; and 3) the psychologist who is onsite for a limited amount of time but provides therapy to a few youth as determined by the staff.

Few studies have addressed health and wellness in the youth population of boarding schools, even though Israel has many youth living in this setting. This research represents the first study that examines all five parameters of youth well-being (including healthy habits, peer relationships, social distress, risky behaviors and school performance) among adolescents residing in Israeli boarding schools. The aims of this study are to:

1. Measure prevalence rates of variables representing well-being among youth residing in an Israeli boarding school.
2. Examine the relationships between variables representing youth well-being and youth perception of their mentor.
3. Assess whether within the Israeli boarding school, there are different subgroups of youth with higher rates of risky and healthy behaviors.

## Methods

This study used a mixed-methods approach. The quantitative portion of the study used existing data consisting of youth responses, which were collected under the auspices of the Ministry of Education to assess health and well-being (n = 158). We received ethics approval for both the qualitative and quantitative portions of this study from the University of Haifa Ethic’s Committee prior to the start of the study. To conduct the secondary data analysis (i.e., the quantitative portion), we obtained and requested Ethic’s Committee approval (#036/13). However, for the qualitative portion of this study, the authors conducted interviews (n = 15) on boarding school staff (for which written informed consent was obtained from the participants and approved by the Ethic’s Committee #278/13). We did not interact with any of the youths in this study. All the participant data were anonymous and provided by the Ministry of Education.

### Quantitative Study

Under the auspices of the Ministry of Education, surveys, using two methods of administration, paper-and-pencil and online, were administered to youth, ages 12 to 18 years (n = 272) who were living at a boarding school in northern Israel. Of those, 58% (n = 158) completed the entire survey. The boarding school decided the days and times to administer the survey. Some students may have been unavailable due to work or being off the campus grounds.

### Procedure and instruments

The nurse within the boarding school entered the height, weight and blood pressure for each respondent into the online survey or on the paper-and-pencil survey. The remaining survey was completed by youth either online or on paper. Surveys were completed by youth in a room reserved for survey completion. The room contained 10 computers and the online survey was anonymous. Surveys (both the online and paper-and-pencil versions) requested no personal identifying information. That is, neither birth date nor gender was obtained to ensure complete anonymity.

The remaining survey was structured with close-ended questions, drawn from among those on the Health Behavior in School-aged Children (HBSC) Survey [43], on the following five areas: (1) health behaviors; (2) school performance; (3) peer relationships and bullying; (4) social distress; and (5) risky behaviors. The HBSC has been used in 43 countries across Europe, Asia and North America [10]. The health behaviors included diet, exercise, and recreational activities such as television and computer use. Questions on diet included a variety of fruits, vegetables, sweets, cola drinks and other common foods, and frequency of consumption (i.e., everyday, 5–6 days per week, 2–4 days per week, once a week, less than once a week or never). Questions on exercise asked how many of the past seven days youth engaged in at least one hour of physical activity. For weekdays and weekends, the survey inquired the number of hours per day of television, computer games, and email or chat room use. For school performance, youth were asked to rank using a Likert scale from very good, good, average or below average, how the youth thinks of him/herself as a student and how the teacher thinks of the youth as a student. Questions on peer relationships included whether the youth had friends, how many and what proportion of friends engage in risky behaviors, all of them, most of them, some of them, few of them or none of them. Social distress was indicated by feeling distressed or being involved in violent activity in the past year with responses of never, a time or two, two to three times per month, at least once a week, or several times per week. Risky behaviors such as smoking, drinking or use of marijuana with responses of never, sometimes, every month, every week and every day.

### Data Analysis

#### Quantitative study

Quantitative data were analyzed using SAS® Version 9.3 and significance was declared at p < 0.05. Most variables were categorical, and comparisons between groups were calculated using Chi-square Tests of Independence. Differences between prevalence rates of youth in this sample versus the general population of Israeli youth were made by comparing odds ratios (OR) and 95% confidence intervals (CI). Cluster analysis was used to identify like groups of youth, those with similar characteristics in the five areas of: healthy habits, peer relationships school performance, social distress, and risky behaviors.

Cluster analysis is a statistical technique that sorts data into relatively homogeneous groups possessing common characteristics. Often cluster analysis is conducted for continuous data by measuring the proximity of values (e.g., Euclidean distance). In fact, there are many cluster analysis techniques for continuous variables. However, in this study, the nature of the survey responses was categorical, and distributions of virtually each variable were bimodal. As a result, variables were recoded into dichotomous variables; and cluster analysis was employed to categorize like groups of youth in the five areas by using the Jaccard coefficient, a robust measure for binary data that calculates the intersection of variables for the number of cases in the union by assigning zero weight to negative matches, and equal weights to positive and non-match responses [44,45].

Due to sample size, a total of two clusters were chosen to represent each area (i.e., healthy habits, peer relationships school performance, social distress, and risky behaviors)–denoting more versus less positive behaviors. For the area of healthy habits, the five variables used to form the clusters included: fruits consumed at least daily, vegetables consumed at least daily, one hour of physical activity at least 5 days/week, and two hours or less of TV watching per day on weekdays, and also on weekends. For school performance, the two variables used to form the clusters included the students’ perception of whether the teacher thinks that the youth was a good student or not and whether the youth thought of him/herself as a good student. For the area of peer relationships, the two variables used to form the clusters included having at least three friends and whether most friends engaged in risky behavior. For the area of social distress, the two variables used to form the clusters included not feeling distressed and not being involved in bullying (as a victim or perpetrator) for the last 12 months. For the area of risky behaviors, the three variables used to form the clusters included smoking either never or rarely, drinking alcohol either never or rarely, and not smoking marijuana in the past 30 days.

#### Qualitative study

In order to better understand the potential causal mechanisms that are associated with our findings, a qualitative component was undertaken as this approach is useful “when researchers are interested in looking beyond identified variables that are statistically linked with a desired effect.” [46] Interviews, using guided conversations, were conducted with boarding school staff (See Table 1). Guided conversation is a well-established technique within quantitative methodology [47]. In these interviews, participants were encouraged to talk about the structure of the boarding school, their vision of the youth’s current and future life and the staff’s goals as their mentors. Participants’ narratives discussing their desires, beliefs, motivation and interpretation lead the interviews to enable a broader understanding of daily life at the boarding school, including the social and personal values of the mentors in within the context of the boarding school.

**Table 1:** **Socio-demographic characteristics of interviewees**

**Gender and age**	**Role**	**Responsibility for grade**	**Education**	**Years worked at boarding school**
Lia, Female 32Y	Mentor	8	14	2
Yoram, Male, 35Y	Mentor	12	16	3
Rina, Female 48Y	Mentor	12	12	6
Merav, Female 46Y	Mentor	9	16	2
Ronen, Male, 38Y	Mentor	10	12	7
Rivi, Female, 53Y	Mother of the House	All	12	23
Rachel, 56Y	Nurse	All	18	8

Interviews were conducted by the first two authors, recorded and then transcribed by author #1. Analysis was thematic and primarily inductive, based on the principles of grounded theory, which enables theoretical insights to emerge from the data [47,48]. Authors discussed achieved consensus on the main themes and obtained confirmation of their validity by the boarding school staff. All the original names were changed to ensure participants’ anonymity.

## Results

The boarding school population contained youth (n = 272) in grades 7th through 12th and ages 13–18 years old (See Table 2). Almost two-thirds were male. Less than half were born in Israel, with a quarter from Ethiopia and a third from countries formerly part of the Soviet Union. More than a quarter were on medication (e.g., Ritalin) for behavior; and slightly less than a tenth was receiving psychiatric care. A third of the youth were categorized as being at level one, almost half were at level two, and less than a fifth were at level 3. Completed surveys were collected only on 58% (158 of 252) youth. Results from analyses of these surveys are shown below.

**Table 2:** **Demographic characteristics of boarding school youth**

	**Youth N = 272**	
**Demographic characteristics**	**Percent**	**(n)**
**Gender**		
Boys	65.8%	(179)
Girls	34.2%	(93)
**Country of birth**		
Israel	41.2%	(107)
Ethiopia	26.2%	(68)
Former Soviet Union	31.3%	(81)
Other	1.6%	(4)
**Taking medication for behavior**	27.9%	(76)
**Receives psychiatric care**	7.4%	(20)
**Level**		
One	34.7%	(87)
Two	46.6%	(117)
Three	18.7%	(47)

**Figure 1:**
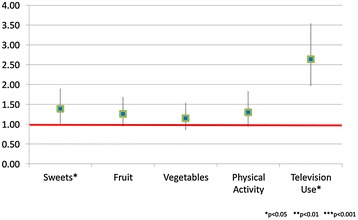
**Health habits among boarding school versus other Israeli youth (odds ratios and 95% confidence intervals).**

Youth living in boarding school had very similar healthy habits compared to other youth living in Israel (See Figure 1). The only differences found were that youth in the general population, compared to those in the boarding schools, were eating more sweets (1.39, 1.02-1.90) and engaging in higher levels of television use (2.64, 1.97-3.54).

Comparisons were made to determine if a positive perception of the mentor was linked to the more positive variable cluster representing the five areas (i.e., healthy habits, peer relationships school performance, social distress, and risky behaviors) (See Table 3). Approximately a third of youth had a positive perception of their mentor. Of the five healthy habits examined, about a third consumed fruits at least daily, vegetables at least daily and participated in at least one hour of physical exercise. Half reported watching two hours or less of television daily or on weekends. These habits did not differ by having a positive perception about their mentor.

**Table 3:** **Behaviors and habits of youth by perception of mentor at the boarding school (n = 158)**

	**Negative perception of mentor (n = 107)**	**Positive perception of mentor (n = 51)**	**Total (n = 158)**
**Behaviors**	**Percent (n)**	**Percent (n)**	**Percent (n)**
**Healthy habits**			
Fruits consumed at least daily	36.5 (39)	37.3 (19)	36.7 (58)
Vegetables consumed at least daily	41.1 (44)	52.9 (27)	44.9 (71)
One hour of physical activity at least 5 days/week	27.1 (29)	31.4 (16)	28.5 (45)
Two hours or less of TV watching per day-weekdays	53.3 (57)	58.8 (30)	55.1 (87)
Two hours or less of TV watching per day-weekends	55.1 (59)	56.9 (29)	55.7 (88)
**School performance**			
Teacher thinks youth is a good student**	59.5 (66)	81.8 (45)	66.9 (111)
Youth thinks of self as a good student**	47.3 (52)	70.9 (39)	55.2 (91)
**Peer relationships**			
At least three friends	62.6 (67)	64.7 (33)	63.3 (100)
Most friends engage in risky behavior	74.8 (80)	88.2 (45)	79.1 (125)
**Social distress**			
Not feeling distressed last 12 months***	39.5 (60)	66.1 (37)	46.6 (97)
Not involved in bullying last 12 months**	36.2 (55)	58.9 (33)	42.3 (88)
**Risky behaviors**			
Smoking – never or rarely	58.6 (65)	50.9 (28)	56.0 (93)
Drinks alcohol- never or rare	32.4 (36)	39.6 (21)	34.8 (57)
Smoked marijuana in the past 30 days – never*	80.8 (84)	94.4 (51)	85.4 (135)

*p < 0.05 **p < 0.01 ***p < 0.001.

Among the two school behaviors, more than half the youth believed their teachers thought they were good students, and youth perceived themselves as good students (see Table 3). Greater proportions of youth with positive perceptions of their mentor believed that the teachers thought they were good students (p < 0.01), and believed themselves to be good students (p < 0.01).

The two social distress measures included feeling or not feeling distressed in the last 12 months and involvement in bullying (as a victim or perpetrator). Almost half reported not feeling distressed in the last 12 months, but only about a tenth reported not being involved in bullying in the last 12 months (see Table 3). As with school behaviors, greater proportions of youth with positive perceptions of their mentor did not feel distressed (p < 0.001), and were not involved in bullying over the past 12 months (p < 0.01).

**Figure 2:**
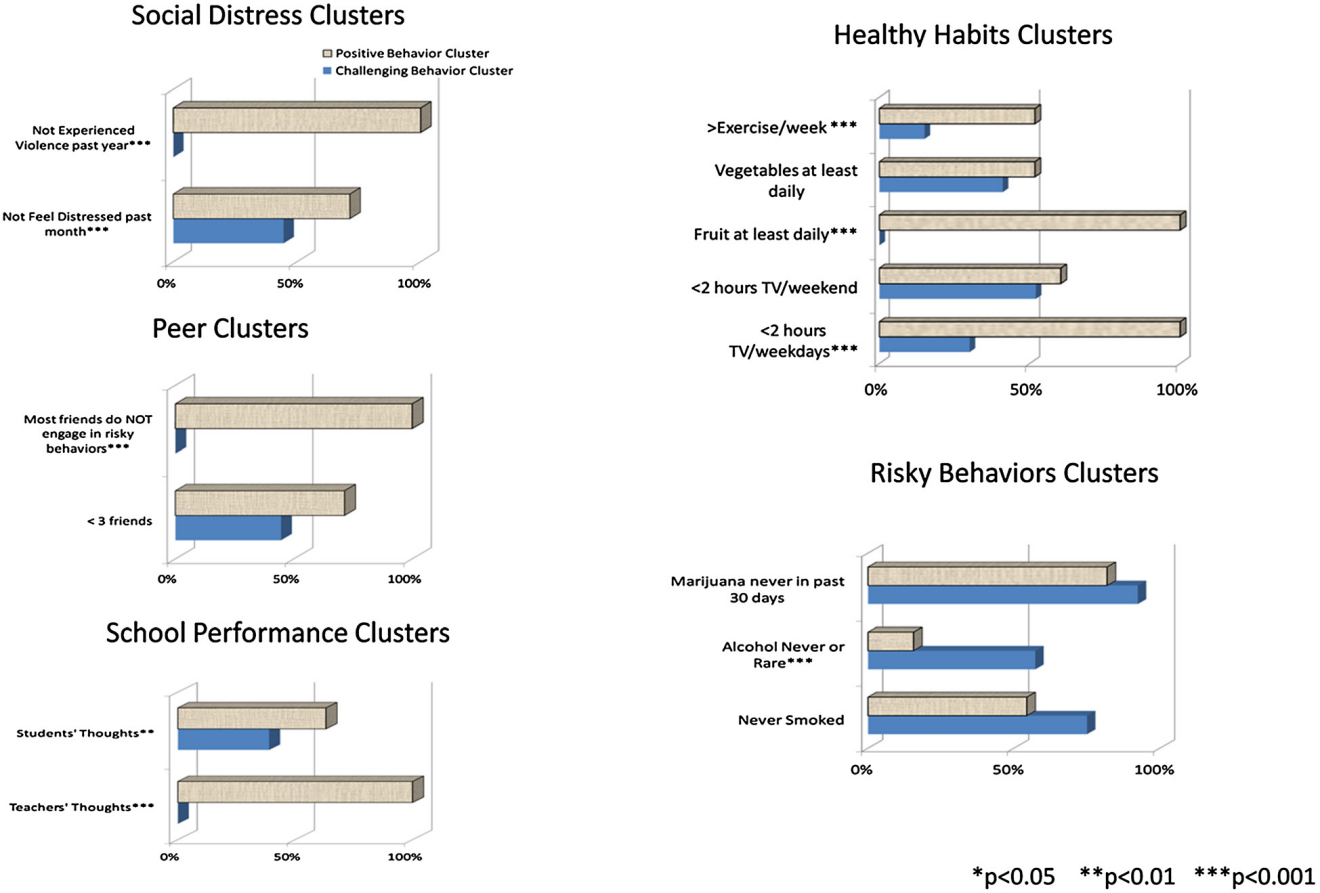
**Clusters: peer relationships, social distress, healthy habits, school performance & risky behaviors.**

Four risky behaviors involving smoking, drinking and using marijuana were measured (see Table 3). Half never or rarely smoked cigarettes. A third never or rarely drank alcohol, and three-quarters had never smoked marijuana. Although there were no differences by perceptions of their mentor for smoking or drinking alcohol, more youth with positive perceptions of their mentor had never smoked marijuana (p < 0.05). Clusters analysis was used to identify like groups or clusters of youth in five areas: healthy habits, peer relationships school performance, social distress, and risky behaviors) (See Figure 2). Of the two clusters that emerged from the variables depicting healthy habits, there were differences between these clusters. Cluster differed by the proportion of youth who exercised at least one-hour five days per week (51.7% versus 15.0%, p < 0.001), ate at least one serving of fruit daily (100% versus 0%, p < 0.001), and watched less than two hours per day of television on weekdays (100% versus 30%, p < 0.001). However, there were no differences between clusters for the following variables: eating at least one serving of vegetables daily and watching less than two hours per day of television on weekends. Peer relationships were depicted by two variables; and in the cluster with the more positive characteristics, more youth reported their friends did not engage in risky behaviors (100% versus 0%, p < 0.001). Clusters did not differ in the proportion of youth who reported having fewer than three friends. School performance incorporated two variables; and in the cluster with the most positive perceptions, more youth described themselves as good students (63.1% versus 38.9%, p < 0.01) and more youth thought their teachers would describe them (the students) as good students (100% versus 0%, p < 0.001). Of social distress’ two clusters, the group with the most positive perceptions were more likely to report not having experienced bullying in the past year (100% versus 0%, p < 0.001) and not feeling distress in the past month (71.4% versus 44.6%, p < 0.001). Of the two clusters obtained from the three variables indicating risky behaviors, the cluster representing the least risky behaviors indicated no or rare alcohol use (15.6% versus 57.3%, p < 0.001). No differences were found between clusters for marijuana use in the past 30 days or ever/never smoked.

Few relationships were found between the perceptions of mentor and the clusters (i.e., healthy habits, peer relationships school performance, social distress, and risky behaviors) (See Table 4). However, an association was found between the clusters of school performance and the youths’ perceptions of mentor (p < 0.001).

**Table 4:** **Clusters by perceptions of mentor**

	**Negative perception of mentor (n = 107)**	**Positive perception of mentor(n = 51)**	**Total (n = 158)**
**Cluster predicting positive activities**	**Percent (n)**	**Percent (n)**	**Percent (n)**
Healthy habit	36.5 (39)	37.3 (19)	36.7 (58)
School performance**	58.9 (63)	80.4 (41)	65.8 (104)
Peer relationships	25.2 (27)	11.8 (6)	20.9 (33)
Social distress	48.6 (52)	62.8 (32)	53.2 (84)
Risky behavior	58.9 (63)	52.9 (27)	57.0 (90)

*p < 0.05 **p < 0.01 ***p < 0.001.

### Boarding school routine

The youth in the boarding school are divided into age groups. Each age group is divided into smaller groups of approximately 15 youth, who are under the supervision of two mentors: a male and a female. These mentors share responsibilities throughout the day. Mentors closely supervise each youth and self-identify as parent-surrogates. They provide emotional support, discipline, guidance, and are confidants. They work with individual youth and groups. Often they are pulled into altercations between youth and provide them with mediation, attempting to teach them to problem-solve for themselves. Also, since youth are from struggling families, mentors assist youth with family issues and crises. The schedule includes: breakfast, school, lunch and variety of after-school activities containing different types of physical activity. Formally, youth are required to choose at least two physical activities per week, although mentors report that most of them do not. The boarding school serves six meals per day following a dietician’s recommendations and based on the World Health Organization’s suggestions of food intake requirements for youth [49]. The boarding school contains all facilities needed for a healthy lifestyle. Qualitative findings with key figures revealed a complicated and multi-faceted picture.

#### “*You can take them out of the neighborhood but you can’t take the neighborhood out of them”*

All the interviewees, especially the mentors described their life with youth as crisis-driven. Most of their energy was invested around disciplinary issues and solving problems that youth brought from their home environment. Yoram, an instructor stated:

*“I know where they come from. I am familiar with their families and their surroundings. You have to consider all these conditions while you work with them. I know what the differences are between the place I grew up and the places where they come from. You can take them out of their neighborhood but you can’t take the neighborhood out of them. We are in a survival mode with them, all the time we have to discipline them and to motivate them to do things such as going to school consistently, things that for regular youth are obvious. You are asking me about physical activity and health eating but we are not there yet…I have to focus on more basic things. Most parts of the day I have to solve conflicts between them or between them and the system.*

#### Social behavior and decent school performance are key elements for better future

School performance and being courteous were noted as key elements for future success. Consequently, the mentors invested their best efforts in these areas. Moreover, the importance of these elements was emphasized as being connected to future army service, an important aspect of Israeli society. (Army service in Israeli-Jewish secular society serves as a symbol for social participation). As stated by Merav, a women, who has worked at the boarding school for two years said:

*“My wish for them is for a future that will change the trajectory of their original life and will give them the chance to fully participate in the society in a good way. I hope they will complete their army service. I think that army service is a predictor for a productive life. In order to increase the odds that they even enter the army they have to complete 12 years of formal education and have good behavior. This is my focus, and it is not easy. These are not the norms in the places from which they come.”*

#### “Smoking is normative here…you have to choose your battles”

The general definition of elements comprising risky behavior is established at the boarding school; and it includes smoking, which is common in all ages of boarding school youth. When we asked the mentors about the reasons for this phenomenon, they explained that they have to choose their battles. The instructors know that most of the youth smoke but the message given youth is that they must smoke in secret. They also distinguish between cigarettes and substances such as alcohol or drugs. As expressed by Rina a woman who has taught at the boarding school for six years:

*“I know that most of them smoke. What can I do? My message for them is that I don’t want to see it; they can't do it in front of any adults. I can smell them and sometimes I comment on that as a joke, but principally, we have to choose our battles. You can’t fight everything, for me school is important, the way they treat each other is important, I would never let them use alcohol or drugs but cigarettes are normal here and you have to face it.”*

#### We don’t have the privilege of having control over their eating habits or physical activity

When the instructors and the mother of the house were asked about healthy eating their answers were usually divided into two themes: theory and practice. In theory, the structure of the boarding school enables and encourages a healthy lifestyle. They serve rich meals that include all food groups and the schedule includes physical activity at least once a day. However, in practice they do not have control over the youth’s choices or the resources to enforce the desired behavior. As stated by the nurse:

*“We serve them according to best practices but we cannot control what they put on their plate. If they prefer to take extra carbs and not vegetables, what can we do? Moreover, we have a group of youth who originally came from Ethiopia, where they used to eat a completely different diet. Here they have discovered the option of chocolate spread with bread and this is their ultimate food preference. The students from Ethiopia are not familiar with this food.”*

## Discussion

Most studies focus on risky activities or mental health behaviors among youth in group homes or residential education (i.e., boarding school) programs. This study employed a more holistic approach and focused on the well-being of boarding school youth in Israel, a country where proportionately more youth reside in boarding schools than anywhere in the world [39]. Well-being was defined by the five parameters of healthy habits, avoidance of risky behaviors, peer relationships, adult relationships, and school environment [10-12]. In contrast to many studies where less exercise and poorer dietary habits were found with disadvantaged youth living in institutional environments [21], this study found that boarding school youth had a lower consumption of sweets and spent less time watching television compared to the general youth population [50]. Consistent with other research, youth who had a positive connection with a mentor reported better school performance [4,9]. However, questions from the internationally-used HBSC survey used to assess healthy and high risk behaviors in youth were insufficiently sensitive to differentiate high from low risk youth groups in this boarding school population.

The boarding school structure may suggest an explanation for both the similarities and differences found in healthy habits. Foods offered in the boarding school are based on policies instituted by the Ministry of Education. Consequently, boarding school youth have at least as good an access to healthy foods as does the general population of youth. However, as with many youth, food choices may not reflect healthy options. Among youth in the boarding school, more than half were from immigrant families; and so, food choices may differ from the general Israeli population. Similarly, since youth rely on the boarding school diet, they may have less access to candies or sweets. Boarding school limits television use, explaining our finding that boarding school youth had significantly lower weekday television exposure compared to youth from the general population. However, note that this difference evaporates for weekend television exposure when youth may return home.

Mentor relationships were associated mainly with school performance, consistent with qualitative findings from the mentor interviews. Interviews revealed that school performance was described by mentors as being the most important predictor of life success. The mentors explained that they “have to choose their battles” with the youth, meaning that they must prioritize their goals, and so, they focus on school performance rather than on physical activity and other health behaviors. Stressing school performance is consistent with the broader Israeli society’s emphasis on education [42]. In fact, school performance is a strong predictor of future success in later life [51,52]. Interestingly, when the mentors were asked about smoking and alcohol use among the youth, they were acutely aware of its prevalence, and mentioned that they had made it clear to youth that these items must be kept hidden from plain view. In this way, mentors made it clear to youth that smoking and alcohol were not encouraged behaviors, but also that they were not focusing on decreasing its use. Yet, no link between positive/negative perception of mentor and youth’s alcohol use was found, despite the finding that alcohol use was the defining variable differentiating high risk and low risk youth. Conversely, mentors were actively encouraging positive school performance. As noted in the definition [6,8,37], mentors in the boarding schools had a consistent and structured relationship, in which they followed the progress of the students. They met weekly with teachers and actively followed school attendance and achievement. Indeed, in Israeli society, school performance also may influence the unit and activities in the mandatory army service, which confers social status in Israeli society [53].

As revealed in other studies [3-5,7,9,36], mentors made a difference in the lives of youth. However, the current study found that it was essential to identify the areas of life that mentors identified as important and to understand the contextual reasons for their beliefs. For example, school performance was seen as vital for success and important within Israeli society, and as a result, the mentors monitored youth progress and worked closely to promote their good school performance. However, mentors did not invest their time or emphasize the importance of healthy habits with youth as a result of limited resources and different priorities.

The HBSC questionnaire has been used in many studies worldwide to differentiate high risk and healthy behaviors [43,54-56]. However, in this study, the questions failed to differentiate youth with high risk behaviors. One potential explanation for the lack of sensitivity is that the questionnaire is designed for the general youth population [43]; and many behaviors, which are considered unacceptable among youth living in relatively safe environments, are considered normative among disadvantaged youth in the group homes and other institutional settings [57,58]. Therefore, this study suggests that the current questionnaire has limitations for at risk youth subgroups.

This study acknowledges its limitations including using a sample from a single boarding school with about a two-thirds representation. This participation rate may be consistent with less generalizability to other populations [59]. Moreover, due to restrictions, more specific demographic characteristics were not elicited. Still, this is among the first studies to examine health behaviors in a holistic way for this unique subgroup of youth who comprise more than 10% of Israel’s high school youth, and who mostly come from disadvantaged families. Although many youth are in residential education programs, group homes and other institutions, the boarding school is a unique structure to Israel; consequently, results may not be generalizable to other residential and vulnerable populations of youth.

## Conclusions

Using a mixed methods design, our study illuminated different perspectives and aspects of boarding school youth in Israel. To the best of our knowledge, this is the first description of healthy and risky behaviors among youth living in an Israeli boarding school. Overall, the behaviors that were compared in this study noted many similarities between youth at boarding schools and youth in the general population. Differences in healthy habits may result from policies governing boarding schools.

Structural elements of residential living may have an influence on its populations. Future research needs to examine more specifically the strengths of the different elements and staff involved in raising youth in non-family residences, particularly the mentors, and the way they shape youth behavior. Mentors have a major influence on school performance among youth, which is the area on which they concentrate. Perhaps if mentors identified healthy habits as an area on which to focus, these habits would also improve.

The well-established HBSC questionnaire used in this study was insufficiently sensitive to distinguish between subgroups of high risk youth. Other reliable and valid tools on healthy and risky behaviors are needed for vulnerable and high risk populations of youth. Adding questions illuminating the influence of mentor and other structural elements found in institutions and residential living on youth would contribute to the dearth of information currently found in this area.
